# Factors associated with unfavourable tuberculosis treatment outcomes in Lusaka, Zambia, 2015: a secondary analysis of routine surveillance data

**DOI:** 10.11604/pamj.2019.32.159.18472

**Published:** 2019-04-08

**Authors:** Francis Hamaimbo Nanzaluka, Sylvia Chibuye, Clara Chola Kasapo, Nelia Langa, Sulani Nyimbili, Given Moonga, Nathan Kapata, Ramya Kumar, Gershom Chongwe

**Affiliations:** 1 Field Epidemiology Training Programme, Lusaka, Zambia; 2 Ministry of Health, Lusaka, Zambia; 3 University of Zambia, Lusaka, Zambia; 4 Zambia National Public Health Institute, Lusaka, Zambia; 5 Zambart TB Project, Lusaka, Zambia

**Keywords:** Surveillance, tuberculosis, treatment, outcome, Lusaka, Zambia

## Abstract

**Introduction:**

Focus has been put on strengthening surveillance systems in high tuberculosis (TB) burden countries, like Zambia, however inadequate information on factors associated with unfavourable TB treatment outcomes is generated from the system. We determined the proportion of tuberculosis treatment outcomes and their associated factors.

**Methods:**

We defined unfavourable outcome as death, lost-to-follow-up, treatment-failure, or not-evaluated and favourable outcome as a patient cured or completed-treatment. We purposively selected a 1^st^ level hospital, an urban-clinic and a peri-urban clinic. We abstracted data from TB treatment registers at these three health facilities, for all TB cases on treatment from 1^st^ January to 31^st^ December, 2015. We calculated proportions of treatment outcomes and analysed associations between unfavourable outcome and factors such as age, HIV status, health facility, and patient type, using univariate logistics regression. We used multivariable stepwise logistic regression to control for confounding and reported the adjusted odds ratios (AOR) and 95% confidence intervals (CI).

**Results:**

We included a total of 1,724 registered TB patients, from one urban clinic 694 (40%), a 1^st^ Level Hospital 654 (38%), and one peri-urban-clinic 276 (22%). Of the total patients, 43% had unfavourable outcomes. Of the total unfavourable outcomes, were recorded as treatment-failure (0.3%), lost-to-follow-up (5%), death (9%) and not evaluated (29%). The odds of unfavourable outcome were higher among patients > 59 years (AOR=2.9, 95%CI: 1.44-5.79), relapses (AOR=1.65, 95%CI: 1.15-2.38), patients who sought treatment at the urban clinic (AOR=1.76, 95%CI:1.27-2.42) and TB/HIV co-infected patients (AOR=1.56, 95%CI:1.11-2.19).

**Conclusion:**

Unfavourable TB treatment outcomes were high in the selected facilities. We recommend special attention to TB patients who are > 59 years old, TB relapses and TB / HIV co-infected. The national TB programme should strengthen close monitoring of health facilities in increasing efforts aimed at evaluating all the outcomes. Studies are required to identify and test interventions aimed at improving treatment outcomes.

## Introduction

Tuberculosis (TB) has continued to be one of the major public health problems and still remains a global emergency for the 21^st^ century [1]. Analysing TB treatment outcomes and their potential risk factors is an important indicator of the performance of a country's TB control program [2]. Monitoring and evaluation of TB patients’ treatment outcomes has been given priority in the End TB Strategy, as an integral part in the treatment and prevention of TB [3]. World Health Organisation (WHO) recommends a 90% TB treatment success rate, which can be achieved through intensified case finding, early diagnosis, availability of medicines, and direct observed therapy in a TB control programme [1]. The estimated TB incidence in 2015 was 391/100,000 population for all ages and treatment success rate of 85% was reported [4]. Many studies have been done looking at factors associated with unfavourable TB treatment outcomes. A retrospective cohort study done in Nigeria found that re-treatment cases, having a positive smear at the second month of follow-up, smear-negative after 2 months of treatment, pulmonary TB, and being male were associated with unfavourable TB treatment outcomes [5]. This study, however, only looked at TB in children. Another study in Mozambique found that being male, having a negative smear result at diagnosis, being HIV positive, and being a retreatment case was associated with unfavourable treatment outcomes [6]. A study conducted in Lusaka [7], found that socio-demographic factors including poverty, being a woman, having extra-pulmonary TB, old age, marital status, and HIV co-infection are associated with unfavourable treatment outcomes.

The burden of TB in Zambia has continued to be high over the years despite the low TB case notification which could be attributed to high rates of undetected TB cases (42%), delayed diagnosis and low utilization of TB preventive therapy (18%) hence seeing unfavourable treatment outcomes above the WHO threshold of less than 5% [8]. Lusaka the capital city of Zambia has some residential areas that came up as a result of rural-urban migration. Apart from this Lusaka also has a number of industries and private businesses which in turn makes a destination centre for people looking for employment and petty trade. This could have led to the increase in the number of TB cases notified for the reason that these people are subjected to poor living conditions [7]. During 2015 a national total of 41588 TB cases was notified, of which 16339 (39%) were notified in Lusaka alone [9]. The afore-mentioned studies indicate that different factors have been associated with TB treatment outcomes in different countries, as well as in different communities within the same country. Therefore, information and identification of factors which affect treatment outcomes in different settings is important to understand contextual problems and accordingly design appropriate community/population-based strategies to reduce the disease burden. In Lusaka district, surveillance data is not routinely analysed to identify possible risk factors that negatively affect treatment outcomes. Therefore, we determined the proportion of factors associated with unfavourable TB treatment outcomes in selected facilities within Lusaka district for the period 1^st^ January to 31^st^ December, 2015.

## Methods

**Definitions of TB treatment outcomes:** Using the WHO definition [10], a TB case was defined as either (1) a bacteriologically confirmed biological specimen positive by either smear microscopy, culture, or Gene Xpert, or (2) clinically diagnosed with TB by a clinician or other medical practitioner who has decided to give the patient a full course of TB treatment. Unfavourable TB treatment outcomes were defined as:

**Death:** A TB patient who died for any reason before starting or during the course of treatment.

**Lost-to-follow-up:** A patient who did not start treatment or treatment interrupted for 2 consecutive months or more.

**Treatment failure:** A patient with sputum smear or culture is positive at month 5 or later during treatment.

**Not evaluated:** Patient who has no treatment outcome reported in the TB treatment register. Thus, it is not clear if a “not evaluated” patient has been transferred out to another treatment unit, or if their treatment outcome is truly unknown.

Favourable TB treatment outcomes include:

**Cure:** A pulmonary TB patient with bacteriologically confirmed TB at the beginning of treatment who was smear- or culture-negative in the last month of treatment and on at least one previous occasion).

**Treatment completion:** A TB patient who completed treatment without evidence of failure BUT with no record to show that sputum smear or culture results in the last month of treatment and on at least one previous occasion were negative, either because tests were not done or because results are unavailable.

We further defined:

**Urban clinic:** TB treatment and diagnostic centre that exists in a properly planned area in the city.

**Peri-urban clinic:** TB treatment and diagnostic centre which exists in an unplanned settlement due to rural urban migration.

**Data collection and variables:** We abstracted variables from the TB treatment registers that were related to the study objectives. Patients' age, gender, health facility type, type of TB, treatment category, and HIV-status were the main explanatory variables. In addition to standard outcome definitions, we classified the final treatment outcome as a dichotomous variable, i.e., favourable outcome (cured or treatment completed) vs unfavourable outcome (death, loss to follow-up, failure or not evaluated) outcomes. We included patients with either bacteriologically confirmed or clinical diagnosed pulmonary tuberculosis (PTB), as well as extra pulmonary tuberculosis (EPTB) and excluded all patients transferred in from other facilities as well as drug resistant TB patients because these forms are reported separately from drug susceptible TB.

**Sampling and sample size:** All the 23 TB diagnostic centres in Lusaka district were put into three categories: hospitals, urban clinics and peri-urban clinics. Based on the anecdotal data from the Ministry of Health, each of these facilities reported more than 300 cases of TB in 2015. We therefore purposively sampled one 1st level Hospital, one urban-clinic and one peri-urban clinic. The sample size was estimated by using the following formula for survey sample estimation at 95% level of confidence.

$$n=\frac{z^{2}p(1-p)}{d^{2}}$$

Where n = the required sample size Z_1-α/2_ = value of the standard normal distribution (1.96) corresponding to a significance level of 0.05 for a 2-sided test. d = margin of error (0.05) p = 0.5 based on the assumption that 50% of the population have unfavourable outcomes due to an unknown prevalence Sample size (n) = 384. Despite having a minimum sample size of 384, we did a complete review and abstraction of all records for confirmed TB cases on treatment during the year 2015 from the standard paper TB treatment registers and verified the recorded information with individual patient TB treatment cards.

**Data analysis methods:** We entered abstracted data from paper-based TB registers into an electronic MS Excel 2010 data entry form and exported to Stata Version 13 analysis software for cleaning and analysis. A histogram was used to graphically check for normality (graphs not shown). The continuous variable age which was normally distributed was categorised to see the various effects in different age categories. Categorical data such as sex was reported using numbers and percentages, chi-square test was used to ascertain associations with unfavourable outcomes. Odds ratios were reported as a measure of association for categorical variables using logistic regression. The proportions were calculated using the number of cases recorded per type of outcome (that is favourable and unfavourable outcome from 1^st^ January to 31^st^ December 2015) data abstracted as the numerator and using the total TB confirmed cases as denominator. Univariate and multivariable logistic regression was performed, the outcome variable was whether or not the individual had unfavourable outcome (treatment failure, died, not evaluated or lost to follow up) or not in 2015. Risk factors assessed include age, sex, location of health facility, HIV status, type of TB, patient type (new, relapse, transfer in or treatment resume case). For the logistic regressions performed using alpha = 0.05, odds ratios and corresponding 95% confidence intervals were calculated for the various risk factors. To keep some priori variables in the model for the multivariable logistic regression, we performed a backward stepwise logistic regression. We subtracted variables from the model using the influence of literature review. Selection of the best model was guided by the likelihood ratio test, Akaike Information Criteria and Bayesian Information Criteria after estimation of the nested models by eliminating variables one at a time. To further understand the treatment outcomes, we performed a multivariable analysis by excluding the not evaluated category from the unfavourable outcomes because, by definition, the real outcome of the patients was unknown. Ethical clearance was obtained from Excellence in Research Ethics and Science (ERES) Ethics Committee (Ref: 2017-June-013), and permission to conduct a study was obtained from the Zambia Ministry of Health.

## Results

**Socio-demographic characteristics and outcome of patients:** We included a total of 1,724 registered TB patients, from the urban clinic 694 (40%), 1^st^ Level Hospital 654 (38%) and peri-urban-clinic 276 (22%) (Table 1). The majority of TB patients were males 1,128 (65.4%), and the age ranged from 0 to 91 years with the majority of patients (51%) aged 30 to 44 years and 985 (57%) had favourable outcomes. Among those with favourable outcomes, 307(18%) were cured and 678 (39%) had completed treatment. The overall treatment success rate for the three health facilities was 57%. We found that 506 (29%) patients were not evaluated, 138 (8%) died, 90 (5%) were lost to follow up and 5 (0.3%) had treatment failure giving an unfavourable outcome of 43%.

**Table 1:** Socio-demographic characteristics and treatment outcomes of the registered TB Cases (N = 1724) at three health facilities in Lusaka District, Zambia, 2015

Characteristic	Urban-Clinic n(%)	1^st^ Level Hospital n(%)	Peri-Urban-Clinic n(%)	Total n(%)	P-Value
TOTAL (n)	694	654	376	1724	<0.001^a^
**Sex**					0.57^a^
Male	445 (64)	430 (66)	253 (67)	1128 (65)	
Females	249 (36)	224 (34)	123 (33)	596 (35)	
**Age group**					0.17^a^
0-14	33 (4)	37 (5)	7 (2)	77 (4)	
15-29	174 (25)	162 (25)	110 (29)	446 (26)	
30-44	358 (52)	324 (50)	192 (51)	874 (51)	
45-59	94 (14)	91 (14)	48 (13)	233 (14)	
> 59	35 (5)	40 (6)	18 (5)	93 (5)	
**OUTCOME**					<0.001^a^
**Favourable**					
Cured	186 (27)	87 (13)	34 (9)	307 (18)	
Treatment Complete	210 (30)	368 (56)	100 (27)	678 (39)	
Favourable outcome [Treatment success rate (%)]	396 (57)	455 (70)	134 (36)	985 (57)	<0.001^a^
**Unfavourable**					<0.001^a^
Not evaluated	173 (25)	117(18)	216 (57)	506 (29)	
Died	51 (7)	67 (10)	20 (5)	138 (8)	
Lost To Follow Up	74 (11)	10 (2)	6 (2)	90 (5)	
Failure	0 (0)	5 (0.3)	0 (0)	5 (0.3)	
Unfavourable outcome (%)	298 (43)	199 (30)	242 (64)	739 (43)	<0.001^a^

a Chi-square test

**Association between demographic/clinical characteristics and treatment outcome among TB patients in Lusaka, Zambia, 2015:** Univariate logistic regression analysis (Table 2) revealed that TB patients above 59 years old had higher odds (unadjusted odds ratio (OR) = 1.65, 95% CI: 1.02, 2.68, p = 0.04) of having unfavourable outcomes compared to those aged 45-59 years, relapse patients had increased odds of unfavourable outcomes (OR = 1.55, 95% CI: 1.22-1.97, p = < 0.001) compared to new patients. Patients who sought treatment at the urban-clinic (OR = 1.7, 95% CI: 1.36-2.14, p = < 0.001) and peri-urban-clinic (OR = 4.1, 95% CI: 3.13-5.36, p = <0.001) were more likely to develop unfavourable outcomes compared to those who sought treatment at the 1^st^ Level referral hospital. Male TB patients were more likely (OR = 1.24, 95% CI: 1.02-1.52, p = 0.03) to develop unfavourable outcomes compared to female TB patients. HIV co-infected TB patients had a statistically significant increased chance of developing unfavourable outcomes compared to HIV negative TB patients (OR = 1.22, 95% CI: 1.00-1.51, p = 0.05).

**Table 2:** Association between demographic /clinical characteristics and treatment outcome among TB patients in Lusaka, Zambia, 2015

Characteristic	Number (%) Favourable Outcomes	Number (%) Unfavourable Outcomes	UOR (95% CI)	P-Value
**Sex**				
Female	361 (61)	235 (39)	1.00	
Male	623 (55)	505 (45)	1.24 (1.02, 1.52)	0.03
**Age group**				
45-59	144 (62)	89 (38)	1.00	
0-14	52 (68)	25 (32)	0.78 (0.45, 1.34)	0.40
15-29	258 (58)	188 (42)	1.17 (0.85, 1.63)	0.32
30-44	484 (55)	390 (45)	1.3 (0.96, 1.75)	0.08
>59	46 (49)	47 (51)	1.65 (1.02, 2.68)	0.04
**Patient Type**				
New	799 (60)	542 (40)	1.00	
Relapse	166 (49)	175 (51)	1.55 (1.22, 1.97)	<0.001
Treatment Resume	16 (44)	20 (56)	1.84 (0.95, 3.59)	0.07
**TB Type**				
Extra Pulmonary	161 (57)	120 (43)	1.00	
Pulmonary	821 (57)	618 (43)	1.00 (0.78, 1.31)	0.94
**HIV Status**				
Negative	351(61)	224 (39)	1.00	
Positive	617 (57)	483 (43)	1.22 (1.0, 1.51)	0.05
**Health Facility**				
1st Level Hospital	356(54)	298 (43)	1.00	
Urban-Clinic	494 (71)	200 (31)	1.7 (1.36, 2.14)	<0.001
Peri-Urban-Clinic	134 (36)	242 (64)	4.1 (3.13, 5.36)	<0.001

CI; Confidence interval; uOR, Unadjusted Odds ratio;

**Analysis of factors associated with Treatment Outcome among TB Patients in Lusaka, Zambia, 2015:** The multivariable logistic regression (Table 3) showed that the odds of unfavourable outcome was significantly higher among relapse patients (aOR = 1.8, 95% CI: 1.42-2.26, p = < 0.001) compared to new patients after adjusting for patient type, location of health facility, age-group, and HIV status. Patients who sought treatment at the urban-clinic (aOR = 1.8, 95% CI: 1.42-2.26, p = <0.001) and peri-urban-clinic (aOR = 4.3, 95% CI: 3.22-5.66, p = <0.001) were more likely to develop unfavourable outcomes compared to the 1^st^Level referral hospital after adjusting for all other factors. The odds (aOR = 1.05, 95% CI: 0.84-1.30, p = 0.67) of unfavourable outcomes were higher among HIV-TB coinfected patients compared to HIV negative TB patients after adjusting for all other factors, the association was however not statistically significant. TB patients aged above 59 years old had higher odds (aOR = 1.8, 95% CI: 1.09, 3.10, p = 0.02) of developing TB compared to those age 45-59 years after adjusting for all other factors. When we excluded the not evaluated category (Table 3) in the multivariable logistic regression we noticed that the odds (aOR = 1.56, 95% CI: 1.11-2.19, p = 0.01) of unfavourable outcomes was significantly higher among the HIV-TB co-infected patients compared to those HIV negative TB patients after adjusting for all other factors.

**Table 3:** Analysis of factors associated with treatment outcome among TB patients in Lusaka, Zambia, 2015

Characteristic	AOR (95% CI)	P-Value	AOR (95% CI) excluding not evaluated category	P-Value
**Age group**				
45-59	1.00		1.00	
0-14	1.11 (0.62, 1.96)	0.73	1.17 (0.50, 2.70)	0.72
15-29	1.2 (0.85, 1.72)	0.28	1.30 (0.77, 2.19)	0.32
30-44	1.4 (0.99, 1.86)	0.06	1.37 (0.80, 2.04)	0.3
>59	1.8 (1.09, 3.10)	0.02	2.90 (1.44, 5.79)	0.003
**Patient Type**				
New	1.00			
Relapse	1.8 (1.42, 2.26)	<0.001	1.65 (1.15, 2.38)	0.007
Treatment Resume	1.5 (0.71, 3.01)	0.31	2.20 (0.88, 5.54)	0.093
**Health Facility**				
1^st^ level hospital	1.00		1.00	
Urban-Clinic	1.8 (1.42, 2.26)	<0.001	1.76 (1.27, 2.42)	0.001
Peri-Urban-Clinic	4.3 (3.22, 5.66)	<0.001	1.05 (0.64, 1.74)	0.834
**HIV Status**				
Negative	1.00		1.00	
Positive	1.06 (0.85, 1.32)	0.62	1.56 (1.11, 2.19)	0.01

CI; Confidence interval; aOR, Adjusted Odds ratio

## Discussion

The present study has highlighted factors associated with unfavourable tuberculosis treatment outcomes among selected health facilities in Lusaka, Zambia, 2015. The study has shown that TB patients that were relapse, HIV co-infected, above 59 years old and those who sought treatment from the urban clinic had increased odds of unfavourable outcomes. The WHO has set standards for reporting of outcome of anti-tuberculosis treatment [10]. Treatment outcomes are a major indicator in the assessment of performance of a national TB programme [1]. In this study, most of the TB patients were males which is similar to findings from other studies [11, 12]. One suggested reason for a higher proportion of males having unfavourable outcomes is that they may be more exposed to TB infections associated with behavioural factors such as ex: smoker, as compared to women [13, 14]. Another possible explanation is that males and females have different societal roles and occupations (such as working in a mine) that can influence not only their risk of exposure to TB but also their access to care [12, 15]. A qualitative study done in Burundi found women to have good patient and self-care in their families which made them more compliant to TB treatment compared to males hence the seen differences [16]. This finding can also be used to explain our findings in this study the difference in study design.

This study found an overall treatment success rate of 57% for the three facilities sampled, and this was lower than the 86% reported for the entire Lusaka district. This finding may mean that overall figures reported in Lusaka district are likely to mask lower treatment success levels in health facilities not performing well. It also suggests that achieving the treatment success target for some health facilities is a major challenge that needs to be tackled. The overall unfavourable TB treatment outcome rate was higher than the unfavourable treatment outcome reported from Ethiopia [2, 17, 18]. This result is also higher than another study conducted in Malaysia which reported a rate of 21.5% [19]. High rates of missing TB cases which stand at 42% and low utilization of TB preventive therapy current at 18% could be contributing to the unfavourable outcome seen in the study [8]. The findings of this study show a high number of cases not evaluated which shows that there is poor record keeping in the three health facilities. The high unfavourable treatment outcome found in this study affects the country's overall goal of achieving the 90% treatment success rate as recommended by the WHO hence there is need to conduct data quality audits to health facilities in order to addressing the challenges faced in data management. Many studies have shown similar to this study that old age is associated with unfavourable outcome in TB treatment [2, 19]. This finding could be attributed to an increased risk of lower immunity, making them more susceptible to infections, as a person grows older which may lead to poor outcomes [2, 20]. One important limitation is that secondary TB register data was used for this study, so we could not control for the age-associated co-morbidities. Findings of a study in Ethiopia [21] are different from what this study found this could be due differences in geographical location also possible confounders due to use of secondary data.

This study also showed that relapse patients had higher odds of developing unfavourable outcomes compared to new cases. Similar to this, a study in India found that relapse cases had a higher risk of developing unfavourable outcomes compared to new cases [22]. This result could be due to many factors, including lack of adherence, higher rates of lost to follow up and higher drug resistance rates among relapse patients [6]. This association could not be shown in this study, because we used secondary data which did not capture these variables. A study conducted in Nigeria did not find any associations between new and relapse patients [23]. In this study, the health facility where a patient was receiving care from was found to be associated with unfavourable outcome. This is similar to a findings from a study done in northern Ethiopia where they compared treatment outcomes from different health facilities [24]. This finding could be as a result of different levels of care received at the facilities. This may include availability of TB diagnostic tools such as Gene-Xpert machines that makes diagnosis easy and fast compared to use of microscopes or culture. It may also be a behavioural factor that could be common in one geographical area of the district, leading to unfavourable treatment outcomes [25, 26]. This is an area that calls for future research to see what risk factors exist in different locations that may put people at higher risk of unfavourable outcomes in the TB treatment. Different from this study, in Nigeria a study compared rural against urban facility and their finding was that patients in rural areas were more associated with unfavourable outcomes [23]. This information tells us there is need to monitor health facilities to learn practices from those that are doing well and trickle them down to those that are not performing well.

The multivariable analysis conducted revealed that after removing the not evaluated category whose outcomes were unknown; HIV positive TB patients were more likely to develop unfavourable outcomes. Similar to other studies [27, 28] our study found HIV positive TB patients to be associated with unfavourable outcome. HIV is known to affect the immune system of individuals making them more susceptible to opportunistic infections such as TB [29]. It has been suggested that HIV positive TB patients are more likely to become malnourished due to constant sickness, have diarrhoea that prevents absorption of nutrients, loss of appetite and sores of mouth that make eating difficult, and also experience more opportunistic infections [14, 23]. Another reason for unfavourable outcomes among HIV positive TB patients could be poor uptake of anti-retro viral therapy (ART) [28, 30]. This study however did not analyse the association between ART and unfavourable treatment outcomes among HIV positive TB patients due to missing information from the registers. This therefore calls for improved action in ensuring that health facilities record and report all TB treatment outcomes. Failure to do so may lead to missing of very important information required for effective service delivery in the diagnosis and treatment of TB. The study was a review of data records in the sampled three health facilities. Socio demographic information such as drinking habits, education levels, marital status and employment [7,24] were not available. Any bias introduced by lack of availability of this data, may have underestimated the association between potential risk factors and treatment outcomes; availability of missing information would have strengthened the association between risk factors and treatment outcomes. Our study purposively sampled three health facilities in Lusaka district. Also the fact that the selection was purposive possible bias might have been introduced which have over or underestimated the true magnitude of the relationship between the exposure and an outcome. This may affect the generalizability of results obtained to the Lusaka district. However, this study was able to identify the potential risk factors associated with unfavourable outcome in the treatment of TB and sampled all the three different categories of TB diagnostic health facilities in Lusaka. These findings may help the national TB programme to adopt effective strategies in the prevention and treatment of TB in Zambia.

## Conclusion

This study has found a high proportion of unfavourable outcomes in three health facilities in Lusaka, which was associated with being > 59 years old, being a relapse case, being HIV positive and receiving care from the urban-clinic. We recommend special attention to patients of > 59 years old, relapse cases and HIV positive receiving TB treatment and also close supervision and monitoring of health facility staff, an effort aimed at evaluating outcomes of notified patients. Studies are required to identify and test interventions aimed at improving treatment outcomes at all levels of health care.

### What is known about this topic

- Unfavourable TB treatment outcomes are associated with being > 59years old, being a relapse case;
- HIV co-infected TB patients are association with unfavourable treatment outcomes.

### What this study adds

- Using routine surveillance data unfavourable outcome are more likely to occur at an urban clinic compared to a 1^st^ level hospital;
- It important to make sure all TB treatment outcomes are evaluated.

## Competing interests

The authors declare no competing interests.
